# Cutaneous leukocytoclastic vasculitis as a nonclassical presentation of Fabry disease in an adult female patient

**DOI:** 10.1016/j.jdcr.2025.09.040

**Published:** 2025-10-11

**Authors:** Carolynne Vo, Jason Solway

**Affiliations:** a University of California, Riverside, School of Medicine, Riverside, California; b Department of Dermatology, Sansum Clinic, Santa Barbara, California

**Keywords:** atypical cutaneous manifestation, Fabry disease, female carrier, leukocytoclastic vasculitis, small vessel vasculitis, X-linked lysosomal storage disorder

## Introduction

Fabry disease is a rare X-linked lysosomal storage disorder caused by mutations in the *GLA* gene, resulting in deficient alpha-galactosidase A activity.^1^^,^^2^ This results in glycolipid deposition, specifically in globotriaosylceramide, in vascular endothelium, kidneys, skin, and peripheral nerves.^1^^,^^2^ Although classically described in males, heterozygous women may develop systemic disease with variable onset and severity from X-chromosome inactivation.^2^^,^^3^

Diagnosis in women may be delayed by normal enzyme levels and the absence of hallmark features such as angiokeratomas or renal dysfunction.^2^^,^^3^ Eventually, most heterozygous female patients develop symptoms often involving the kidneys, heart, or central nervous system, about a decade after affected males.^3^^,^^4^ Genetic testing is essential in suspected cases, especially when clinical signs are atypical.

Fabry disease frequently mimics autoimmune or inflammatory conditions such as lupus, rheumatoid arthritis, or vasculitis.^1^^,^^2^ While angiokeratomas are the most recognized dermatologic sign, women with Fabry disease can also present with atypical skin findings, including vasculitic purpura.^4^^,^^5^ In such cases, dermatologic signs represent a valuable window into an otherwise elusive systemic disorder.

This case describes a nonclassical cutaneous presentation initially diagnosed as leukocytoclastic vasculitis (LCV) in a female patient. The eventual diagnosis of Fabry disease emphasizes the importance of considering metabolic and genetic etiologies when vasculitic lesions arise without definitive autoimmune markers. Cutaneous purpura may also represent vasculopathic injury with or without superimposed leukocytoclastic changes.

## Case report

**Fig 1:**
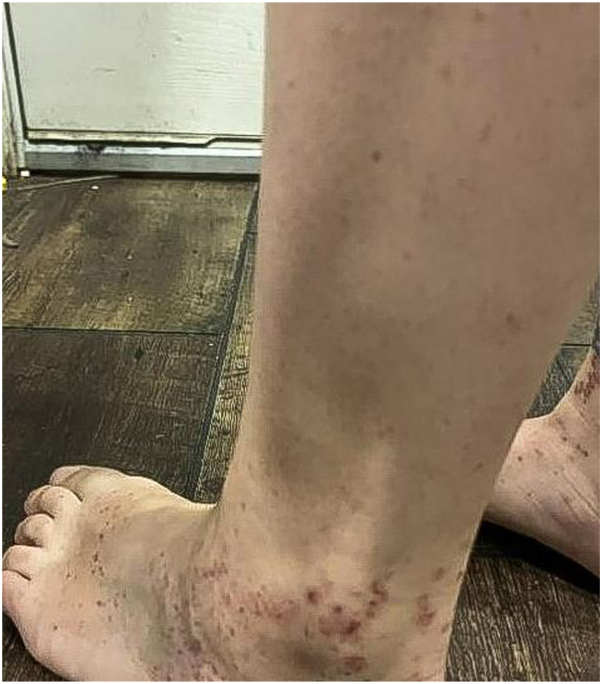
*Initial presentation of violaceous, nonblanching purpura on the posterior ankles.* The lesions were tender to palpation and appeared without ulceration.

**Fig 2:**
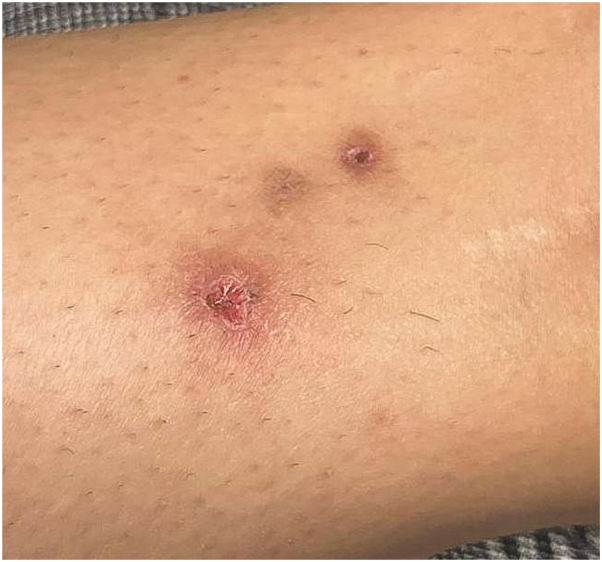
*Progression of skin papules with central ulceration and surrounding erythema on the lower extremity.* Over time, the purpuric lesions evolved into blistering, ulcerated papules.

A 36-year-old female with no prior medical history presented in March 2024 with a painful, burning rash that began around her ankles and spread to her lower extremities (Fig 1). The lesions gradually progressed into blisters, which opened into ulcerative papules (Fig 2). Initial workup, including CBC, CMP, ESR, CRP, ANA, and RF, was unremarkable. A short course of prednisone (60 mg/day) led to partial improvement, but the rash persisted distally.

She reported chronic joint pain and ankle swelling, without systemic, gastrointestinal, or mucocutaneous symptoms. Family history included immune thrombocytopenic purpura in her mother.

In September 2024, she returned with acute sinus pressure, right-sided hearing loss, and a facial rash. ED documentation described an urticarial, tender rash with a normal chest X-ray and laboratory workup. She was discharged with azithromycin and a prednisone taper (40 mg/day), resulting in clinical improvement of her facial rash.

By October 2024, urinalysis revealed microscopic hematuria and mild proteinuria. Additional labs showed elevated total protein and beta-globulin levels, and positive thyroid peroxidase antibodies. Rheumatology was consulted due to concern for systemic vasculitis. Imaging studies included sinus computed tomography, which showed bilateral mucosal thickening, and a normal chest computed tomography.

Dermatology was involved in November 2024. A punch biopsy from the right calf confirmed LCV. However, direct immunofluorescence (DIF) was negative. Although systemic symptoms improved, hematuria and proteinuria persisted, prompting nephrology referral.

A renal biopsy in March 2025 revealed IgA-dominant immune complex glomerulonephritis with podocyte inclusions and myelin figures. CRP was also noted to be elevated (12.6 mg/L). Both findings raised suspicion of Fabry disease. Genetic testing subsequently identified a pathogenic heterozygous *GLA* mutation, confirming X-linked Fabry disease.

## Discussion

This case illustrates a diagnostically challenging presentation of Fabry disease in a female patient, initially manifesting as LCV. Although angiokeratomas and anhidrosis are hallmark cutaneous findings in Fabry disease, emerging literature describes atypical dermatologic manifestations that may delay diagnosis.^1^^,^^5^ Persistent or recurrent vasculitic lesions, especially when painful or refractory to treatment, should prompt consideration of metabolic or genetic disorders.

LCV is an immune complex–mediated, small-vessel vasculitis characterized by neutrophil infiltration and fibrinoid necrosis in postcapillary venules.^6^ It presents with palpable purpura and typically triggers evaluation for autoimmune, infectious, or drug-related etiologies.^6^ Her constellation of LCV, joint pain, and proteinuria resembled an autoimmune process. However, negative serologies and DIF results made autoimmune etiologies less likely. We acknowledge that the diagnostic yield of DIF is influenced by lesion age and biopsy timing, and a negative DIF does not exclude immune complex–mediated disease. The persistence of unexplained purpura and renal abnormalities ultimately led to renal biopsy. Nonetheless, histologic features such as lipid-laden vacuolated cells in dermal blood vessels and sweat glands can help reinforce clinical suspicion of Fabry disease, especially before confirmatory genetic testing.^7^

Disease expression in female patients is highly variable due to X-chromosome inactivation.^3^

When lyonization favors the mutated *GLA* allele, symptoms can resemble those in affected males.^3^ Yet, many women remain undiagnosed until overt organ damage occurs or are only identified through family-based genetic screening.^3^^,^^4^ In 1 cohort, 85% of affected women were diagnosed via cascade testing, and only 38.5% were symptomatic at the time.^3^^,^^4^

In our patient, the absence of hallmark signs such as angiokeratomas, corneal verticillata, or acroparesthesias contributed to diagnostic delay. Given Fabry’s underlying vasculopathic mechanism, purpura may reflect vascular injury in the presence or absence of true LCV and complicate histopathologic interpretation.^7^ Skin biopsies in female patients typically show reduced globotriaosylceramide deposition compared to males, which limits diagnostic utility.^7^

Renal biopsy provided the key diagnostic clue: IgA-dominant glomerulonephritis with myelin figures in podocytes, consistent with Fabry nephropathy.^8^ Notably, the coexistence of IgA-dominant immune complex glomerulonephritis could represent an atypical manifestation of Fabry’s or a concurrent IgA vasculitis, as has been rarely described.^7^ This finding, combined with persistent hematuria and proteinuria, prompted genetic testing and confirmed diagnosis.^8^^,^^9^

This case highlights key diagnostic considerations in women with Fabry disease. First, Fabry disease may present with non-classic dermatologic features. Second, normal enzyme activity does not exclude the diagnosis due to variable X-chromosome inactivation, making genetic testing essential.^9^ Third, tissue biopsies of skin or kidney offer valuable clues, especially when autoimmune workups are inconclusive. Finally, early diagnosis allows timely initiation of enzyme replacement or chaperone therapy and facilitates family screening.^9^^,^^10^

Despite advances in diagnostic tools and therapies, female patients remain underdiagnosed and undertreated.^3^^,^^4^ A more proactive workup in women involving earlier consideration of Fabry disease with unexplained purpura and renal findings may prevent irreversible organ damage.

This case underscores the vital role of dermatologists in recognizing cutaneous signs and the importance of a multidisciplinary evaluation in identifying rare systemic disorders. Early recognition of inconclusive workups and atypical symptoms can enable timely intervention.

## Conflicts of interest

None disclosed.
